# AI in the EP lab – mapping, imaging, and signal interpretation

**DOI:** 10.1016/j.ipej.2026.05.003

**Published:** 2026-05-26

**Authors:** Abhinav B. Anand, Ketan Rajawat

**Affiliations:** a Department of Cardiology, Narayana Health, Mysuru, India; b Department of Electrical Engineering, Indian Institute of Technology Kanpur, Kanpur, Uttar Pradesh, 208016, India

**Keywords:** Artificial intelligence, Electrophysiology, Machine learning, Atrial fibrillation, Ventricular tachycardia

## Abstract

Artificial intelligence (AI) is the use of computational models to learn from electrical, anatomical and imaging data to assist or automate interpretation, prediction and decision making in arrhythmia diagnosis and treatment. This includes interpretation of signals, integration of multimodal data to support procedural decisions and to predict outcomes. When personalized to individual patients, these mechanistic approaches give rise to cardiac digital twins capable to procedural planning and hypothesis testing.

In the electrophysiology (EP) lab, AI aims to reduce inter-observer variability, improve identification of the arrhythmogenic substrate by combining information from multimodality imaging and promises to streamline mapping and ablation workflows. Integration of AI with cardiac CT and cardiac MRI allows for automated segmentation, wall thickness and scar characterization and identification of conducting channels for ventricular tachycardia. Similarly, AI guided approaches for spatiotemporal dispersion and focal drivers in atrial fibrillation have demonstrated improved procedural consistency and promising clinical outcomes.

This review synthesizes the current evidence on use of AI in the EP lab particularly preprocedural planning, intra procedural imaging and use of digital twins. We highlight practical workflows, representative clinical use cases and key limitations of AI.

## 1 Introduction

**Fig. 1:**
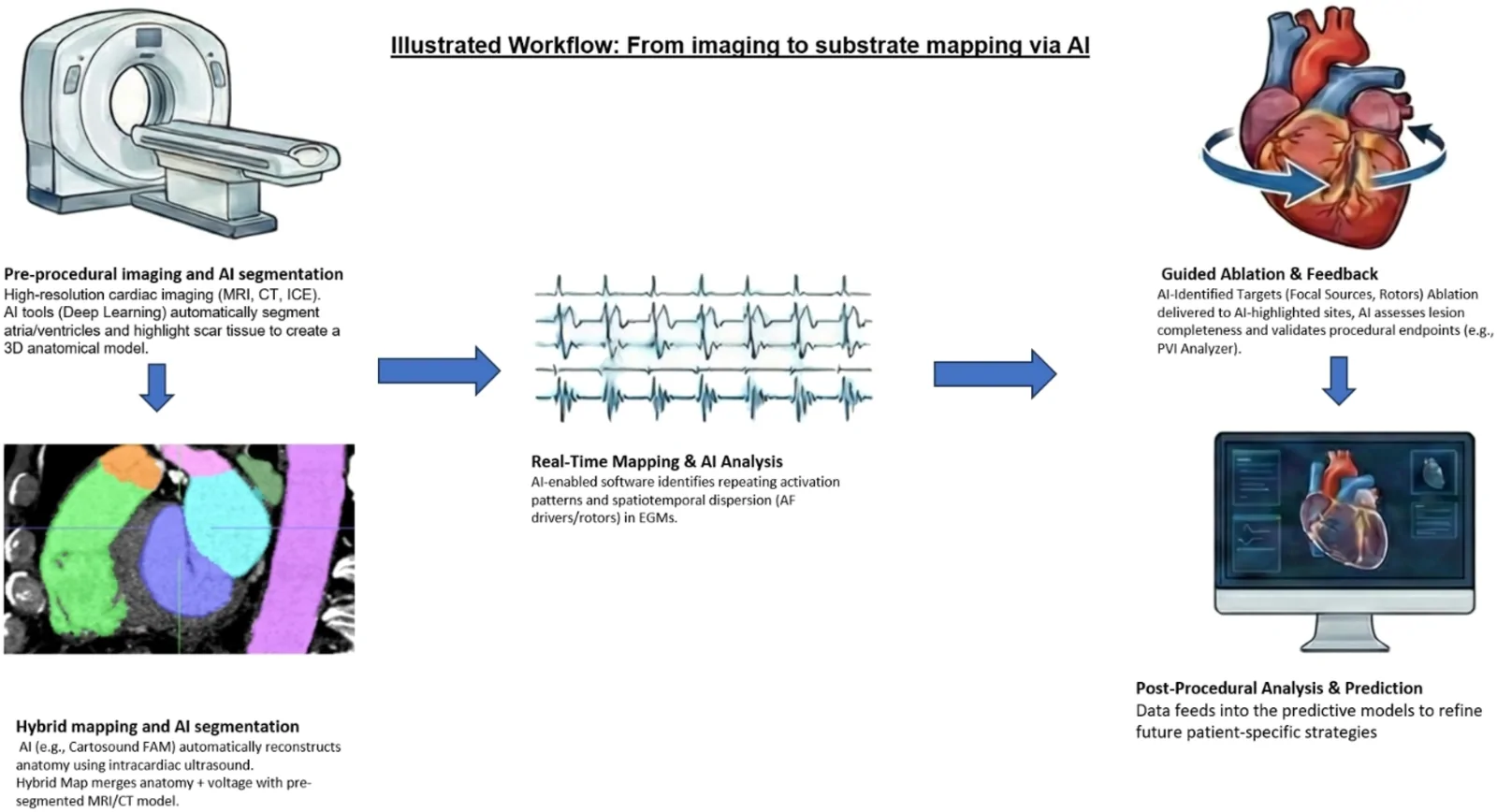
Illustrated Workflow: From imaging to substrate map via AI.

Artificial intelligence (AI) in electrophysiology (EP) can be viewed as a spectrum of methods with two complementary ends. At one end are data-driven models that learn patterns directly from data (for example, electrograms, ECGs, images, or clinical variables) to classify signals, identify substrate, or predict outcomes. These methods can be highly effective but may be sensitive to how data are acquired, labelled, and represented, and their performance can degrade when applied to new centres or different patient populations [1]. At the other end are mechanistic models, which simulate cardiac electrical behaviour using biophysical principles. When such mechanistic models are made patient-specific using an individual's anatomy and substrate, they form a cardiac digital twin that can be used for diagnosis and procedure planning. In practice, these approaches are increasingly hybrid, with modern digital twins using both data-driven steps as well as mechanistic simulations [2]. [3]Creating a digital twin of individual patients based on computational modelling allows simulations to be performed without subjecting the patient to the procedural risks. This is particularly important for patients with complex arrhythmia substrate and arrhythmias associated with hemodynamic instability where this promises to reduce the procedural time in an effective way and has the potential to change the mapping and ablation workflows (Fig. 1). The use of AI can be divided into pre-procedure, intra-procedure and post-procedure parts. We will be concentrating mainly on the first two parts in this review.

## 2 Signal processing

Reliable signal interpretation forms the foundation upon which electrophysiology studies are carried out. A limitation of traditional bipolar mapping which measures the voltage difference between two adjacent electrodes is the directional sensitivity of bipolar electrodes leading to bipolar blindness. This is because, if the wavefront travels perpendicular to the electrode pair, it arrives near simultaneously at both the electrodes resulting in a net voltage difference of near zero. This can lead to erroneous calculation of healthy tissue as scar.

### 2.1 Algorithmic derivation of true electrogram

Abbott's EnSite system utilizing the Advisor HD Grid catheter uses Omnipolar Technology (OT) to solve this geometric problem. The HD grid mapping catheter utilizes an array of 16 electrodes of 1 mm length in a 4×4 square lattice with equal inter-electrode anatomical distance. The adjacent orthogonal bipolar pairs form a triangular group called cliques which is then used to determine the electrophysiological properties. Using bipolar signals along specific directions and signal processing, maps are generated in real time from a single depolarization. This is independent of the catheter wavefront orientation [4].(Table 1).

**Table 1:** Commercial AI-enhanced mapping systems with capabilities.

System	Manufacturer	Key AI/Algorithmic Feature	Primary Function & Mechanism	Data Source	AI
EnSite X	Abbott	Omnipolar Technology (OT)/OT Near Field	Signal Fidelity: Solves "bipolar blindness" by calculating the EGMs from electrode cliques. OT Near Field uses frequency analysis to mathematically isolate local activation from far-field bystander signals.	Intracardiac EGMs (HD Grid)	Clique-based signal processing
CARTO 3 (V8)	Biosense Webster	CARTOSOUND™ FAM Module	Automates the creation of the LA shell from ICE. Uses a DL model trained on ultrasound images to identify endocardial borders and reject artifacts.	Intracardiac Echo (ICE)	Deep Learning (Convolutional Neural Networks: CNNs)
Rhythmia HDx	Boston Scientific	LUMIPOINT/Skyline	Automates the detection of late potentials, fractionation, and critical isthmuses by analysing signal histograms and timing windows.	Intracardiac EGMs (Orion)	Advanced algorithms
VX1	Volta Medical	VX1 Dispersion Algorithm	Substrate Identification: Identifies "spatiotemporal dispersion" (AF drivers). Uses a supervised DL model trained on expert-annotated EGMs to classify signals as "dispersed" or "non-dispersed."	Intracardiac EGMs (Multipoint)	Supervised Deep Learning

The standard of electrogram timing annotation is based on the maximum negative slope (-dV/dT) for local activation time. While this works for presence of normal conduction through healthy myocardium, this is substantially inferior in presence of complex fractionated and low amplitude electrograms. The focal source location error is significantly lower with better consistency for the Omnipolar maps compared to the traditional bipolar maps. Similarly, the near field signals can be analysed by using peak frequency which detects signal sharpness irrespective of the amplitude to separate the near field from far field potentials. EnSite X EP system also allows selection of EGM annotation based on onset or offset of the signal, allowing mapping the earliest site during focal tachycardia and targeting the areas of slow conduction in abnormal substrate with late potential annotation [4,5].

The Lumipoint algorithm is able to detect all the activations present in every electrogram. It detects these irrespective of the local activation time and automatically highlights areas with electrograms having specific characteristics or timings. With the help of specific windows of interest, adjusting to the post QRS phase, it can help find late potentials. In ventricular tachycardia, the presence of fractionated or multiple late potentials can be automatically annotated using the complex activation search feature. The region of map corresponding to minimum activating tissue may identify the putative isthmus of a VT circuit, though this may also occur with blind loops, so entrainment is still required to differentiate these regions [6]. (Table 1).

The SKYLINE graph on the Lumipoint algorithm, which reflects the size of activated region, can be used to display the decrease in activation area which indicates areas of slow conduction. This can be subsequently highlighted in the isochronal map, which can be then used to see the wavefront property and characteristics of local signals in the area. In a study by Li et al., successful ablation sites were located in areas with GAH valley <0.2 and 0.2-0.4 [7]. (Table 1).

## 3 Atrial fibrillation

Atrial fibrillation (AF), the most common heart rhythm disorder, is associated with the risk of stroke, heart failure, cardiovascular hospitalization and mortality [8]. The standard treatment of AF is pulmonary vein isolation (PVI), however, PVI only ablation has a success rate of only 59-71 percent in patients with persistent AF (PsAF) [9].In patients with PsAF, the atria remodel and become fibrotic which creates slow and disorganized conduction resulting in possible reentry. However, use of extra PVI ablation strategies have not consistently resulted in improved procedural and long-term success [10,11]. A key challenge has been to ensure objectivity, consistency and reproducibility across various centres.

Despite significant advances in recent technology, recurrence rates for complex cases remain unacceptably high. While standardized treatment approaches work in few patients, high recurrence rates demand an individualized approach to account for the variability in the individuals [12].

### 3.1 Utilization of CT for procedure planning

Multidetector computed tomography has been used to characterize the left atrium (LA), pulmonary vein (PV) anatomy and to rule out LA clot prior to atrial fibrillation ablation procedures. Use of data from the MDCT scans to detect the LA wall thickness and to adapt the ablation index to left atrial wall thickness (LAWT) to determine transmurality has been shown to be feasible, effective and safe; resulting in shorter procedures, fluoroscopy and RF times. By adapting ablation index values to the LA wall thickness, the RF requirement to achieve the first pass isolation was significantly lower, though image segmentation step resulted in additional image processing time of 15±2min [13].

The CARTOSEG CT module utilizes trained neural network to identify and automatically segment cardiac structures from a preprocedural CT scan. It identifies the heart chambers and constructs 3 D meshes that can be instantly registered to the real time EAM. This automation reduces the segmentation task to mere seconds, streamlining the workflow [14]. [15].

Similarly, non-pulmonary vein triggers have been predicted using deep learning on the pulmonary vein CT. Since these can predict the triggers prior to the ablation based on the cardiac structure, this may reduce the rate of atrial fibrillation recurrences [16].

### 3.2 Machine learning (ML) and Deep learning (DL) to identify focal triggers

The use of ML and DL to learn and automate patterns in detection of arrhythmia substrate triggers and ablation signals has the potential to reduce interobserver variability in identification of ablation targets as well as to predict the ablation strategy. Use of deep learning has also been used to identify focal source and trigger (FaST) to automate focal sources detection by detecting sustained periodic unipolar QS electrogram detection. FaST ablation terminated AF in 30% of patients, and reduced AF recurrence by 48% at 1-year follow-up, although this was defined based on single recording site and not on activation pattern from a multielectrode array [17].

### 3.3 Real time guidance and integration

Local clusters of electrogram, with time and space dispersion of activation; forming a localized sequential activation in 3/more adjacent bipolar electrogram; spread over the entire AF cycle have showed to corelate with high degree of acute termination of atrial fibrillation with low level of recurrence. The dispersion-based ablation to target these local drivers is difficult to do visually and VX1 software by Volta has been trained to detect these areas. In the initial series of 4 patients, of which 2 were redo ablations, VX1 was used for real time adjudications of multipolar electrograms with satisfactory acute and medium-term outcomes [18].

Use of Volta VX1 to guide catheter ablation of spatiotemporal dispersion detected by AI guided software in patients with persistent AF resulted in a freedom from AT/AF of 78 percent after a single procedure and of 82 percent after a total of 1.46 ± 0.68 procedures. Similar to other approaches for substrate based ablation, the authors consider DISPERS ablation as a stepwise sequential ablation approach to achieve long term arrhythmia freedom [19].

Tailored AF was a multicenter randomized trial used AI based Volta AF-Xplorer™ software to compare AI guided ablation in addition to PVI to a conventional PVI only procedure. One year post procedure the trial met its primary efficacy endpoint in 88 percent of the patients in tailored arm compared to 70 percent of the patients in the anatomical arm (p < 0.0001). Use of AI in this trial led to objective, reproducible and reliable identification of ablation target areas across all 26 centres. Although the primary end point only focuses on freedom from AF, and not any atrial arrhythmia, AT recurrences are easier to ablate and can be seen towards a step to sinus rhythm [20]. (Table 1).

Similarly, STAR (Stochastic Trajectory Analysis of Ranked Signals) mapping, uses data on multiple individual wavefront trajectories to identify leading areas which precede activation of neighbouring areas. Using MATLAB software and data from unipolar electrograms, chamber geometry and catheter electrode location data, STAR maps were created to generate local drivers and subsequently targeted. Patients undergoing ablation by STAR mapping were more likely to achieve atrial fibrillation termination as a procedural endpoint and higher rates of freedom form AF/AT during long term follow-up with fewer procedures long term [21].

Post procedure, data from the case can be uploaded and stored anonymously on the cloud using CARTONET. This can be remotely viewed to review the procedure, ablation analysis and for workflow optimization. AI based ablation analysis also provides retrospective insights with respect to potential reconnections, anatomical segmentation and ablation index [22].

### 3.4 Digital twins

A cardiac digital twin is a patient-specific computational model designed to integrate anatomy, substrate, and electrical behaviour into a single framework that can be interrogated before and alongside an EP procedure. The practical goal is to move from describing substrate to testing hypotheses such as which circuits are plausible in this patient, where sustaining pathways are likely to exist, and how different lesion sets might prevent re-entry while minimizing unnecessary ablation. Constructing a digital twin typically begins with high-resolution imaging (CT and/or MRI) to define chamber geometry and structural abnormalities such as scar or fibrosis. These images are segmented to delineate relevant cardiac structures, then converted into a three-dimensional computational mesh. When non-invasive body-surface signals are available, alignment between the cardiac anatomy and torso geometry supports simulation of ECG-level outputs that can be compared with measured recordings. Substrate information from imaging (for example, intensity-based scar representations on LGE-CMR or CT-derived surrogates) is mapped onto the mesh, and model parameters that govern electrical propagation are assigned [23].

Once assembled, the model can be used for in-silico experiments. For instance, pacing and stimulation protocols can be applied virtually to probe arrhythmia inducibility, identify regions that repeatedly participate in sustaining propagation, and estimate candidate pathways consistent with re-entry. Virtual lesions can then be applied to test competing ablation strategies, allowing prioritization of targets before extensive invasive mapping is performed. This approach is particularly attractive when mapping is limited by hemodynamic intolerance, because it can generate a patient-specific shortlist of regions to confirm in the lab rather than requiring an exhaustive search.

In a study by Trayanova et al., LGE MRI were used to construct digital twins of the patients’ atria reflecting the fibrosis distribution, from which maps of fibrosis density and fibrosis entropy were constructed. The arrhythmogenic propensity was then assessed by stress testing it virtually. Two types of rotors were identified during stress testing: location of independent rotors (LIRs) and dependent rotors which could be eliminated after ablating elsewhere in the substrate (location of contiguous rotors). The substrate locations were then targeted by virtual lesions and inducibility was repeated till the substrate became non inducible. Use of this strategy with a goal to give minimum lesions while reducing the chances of redo procedure, resulted in a substantial reduction in the number of lesions by 34 %; while at the same time preventing iatrogenic atrial tachycardia (iAT)) by 47% [24].

Similarly, in the OPTIMA study, in patients with persistent AF, based on the fibrotic substrate, an optimal set of ablation lesion was determined which would fully ablate the arrhythmogenic propensity of the atrial substrate. In a proof-of-concept feasibility study, PsAF did not recur in any of the patients, and only one patient underwent ablation in the follow-up period for paroxysmal AF/atrial flutter [25].

A key distinction between a generic simulation and a true digital twin is personalization, defined as the process of tuning the model so that its outputs resemble the patient's measured electrical behaviour. Conceptually, personalization is an iterative loop since the model is simulated forward to generate predicted activation or ECG patterns, which are compared with measured data and uncertain parameters are adjusted to reduce mismatch. Repeating this cycle progressively calibrates the model toward the individual, improving the relevance of predicted drivers, pathways, and responses to virtual lesions. Importantly, this is also a natural interface between AI and mechanistic modelling since efficient optimization strategies and learned surrogate models can reduce the number of expensive simulations needed to reach an acceptable fit, making personalization more feasible for clinical workflows.

## 4 Ventricular tachycardia

### 4.1 Imaging workflows: utilization of MRI/CT for procedure planning and ablation for ventricular tachycardia

Although the surface ecg is a reliable marker of the exit site of the ventricular tachycardia, the target of the ablation is the isthmus, which can be hard to localize due to complex 3D circuits and bystander areas [26]. [27] This is compounded by the fact that patients with structurally abnormal heart and scar VT are sicker and hemodynamically unstable VT is not well tolerated by the patient. The patients with VT are complex and may have non ischemic cardiomyopathy on the background of a coronary artery disease or dual cardiomyopathy [28]. Long procedures increase risk to the patient as well as to the operator. Success rates for eliminating VT are modest especially in the non-ischemic cardiomyopathy sub group [29].

To reduce the duration of the procedure, the preprocedural imaging helps to visualize the arrhythmogenic substrate before the procedure begins. Beyond visualization, patient-specific modelling extends preprocedural imaging into a virtual testing environment, allowing candidate pathways and ablation strategies to be explored in silico before time-limited mapping in the lab.

Wall thickness in post myocardial infarction patients is heterogeneous. Channels of preserved thickness are bordered by thinner scar, since areas of severe thinning are indicative of dense scar acting as a zone of conduction block and VT isthmuses will be in the channels of relatively preserved thickness. Wall thickness has been shown to co-localize with voltage defined scar. This information provides a more specific and clearly defined targets in patients with ventricular tachycardia. Scar distribution by LGE- CMR also decides the best approach to reach the whole substrate. MRI has also been used to locate scar tissue by utilizing pixel signal intensity maps from the late gadolinium enhancement -CMR to allow direct targeting of the heterogeneous tissue channels.

**Fig. 2:**
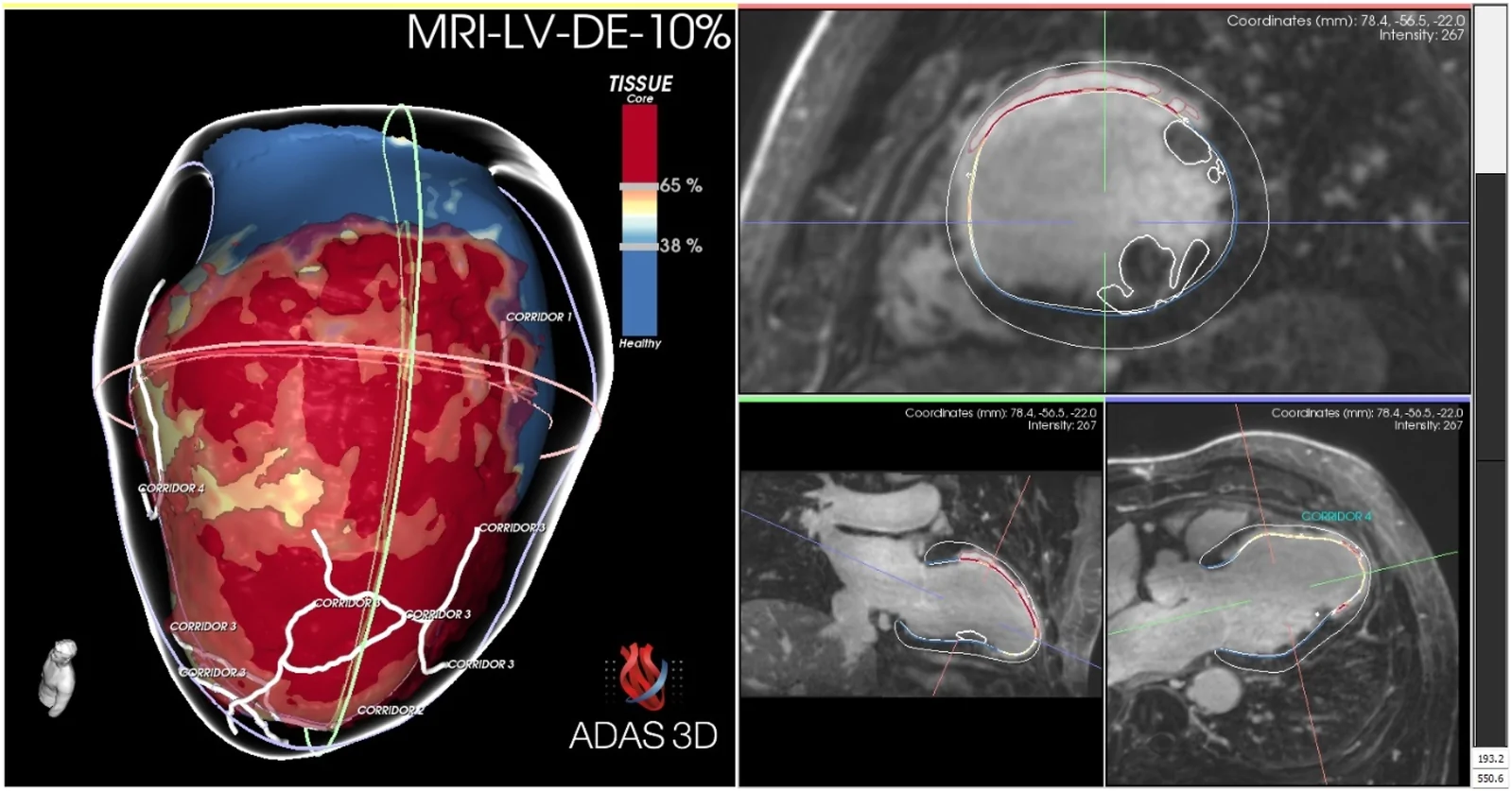
Use of ADAS 3D software to facilitate identification of target ablation site(s). (Left) Pixel signal intensity (PSI) map from late gadolinium enhancement-cardiac magnetic resonance (LGE-CMR) study is used to detect heterogenous tissue channels and guide the ablation strategy. (Right) (Image courtesy of ADAS 3D Medical SL, Barcelona, Spain.).

In a study by Soto-Iglesias et al., heterogeneous tissue channels were defined as continuous corridors of border zone surrounded either by scar or anatomical barrier. These were classified as subendocardial, transmural or epicardial. This also guided the access strategy during ablation. In patients who had scar area of >14 cm^2^ in the epicardial layer, the epicardial access was undertaken. Preprocedural imaging by CMR and guiding ablation directly with CMR derived targets halved the total procedure times and resulted in more complete substrate elimination and better long-term freedom from VT [30]. CMR derived pixel signal intensity (PSI) maps are particularly useful in identifying the conducting channels in the border zone between dense scars and healthy ones. This is, sometimes, not well defined by electroanatomic mapping (EAM) due to far field effect of surrounding healthy myocardium and lack of sensitivity to analyse local electrograms (EGMs) deeper than the endocardium due to far field effect of the surrounding tissue (Fig. 2).

While MRI has been useful in detecting the channels based on late gadolinium enhancement, there are significant limitations to this, especially in patients with chronic kidney disease and claustrophobia [31].Patients with cardiac implanted devices will also generate artifacts which decreases the quality of images obtained through the MRI. A workaround to this problem is the use of cardiac CT.

**Fig. 3:**
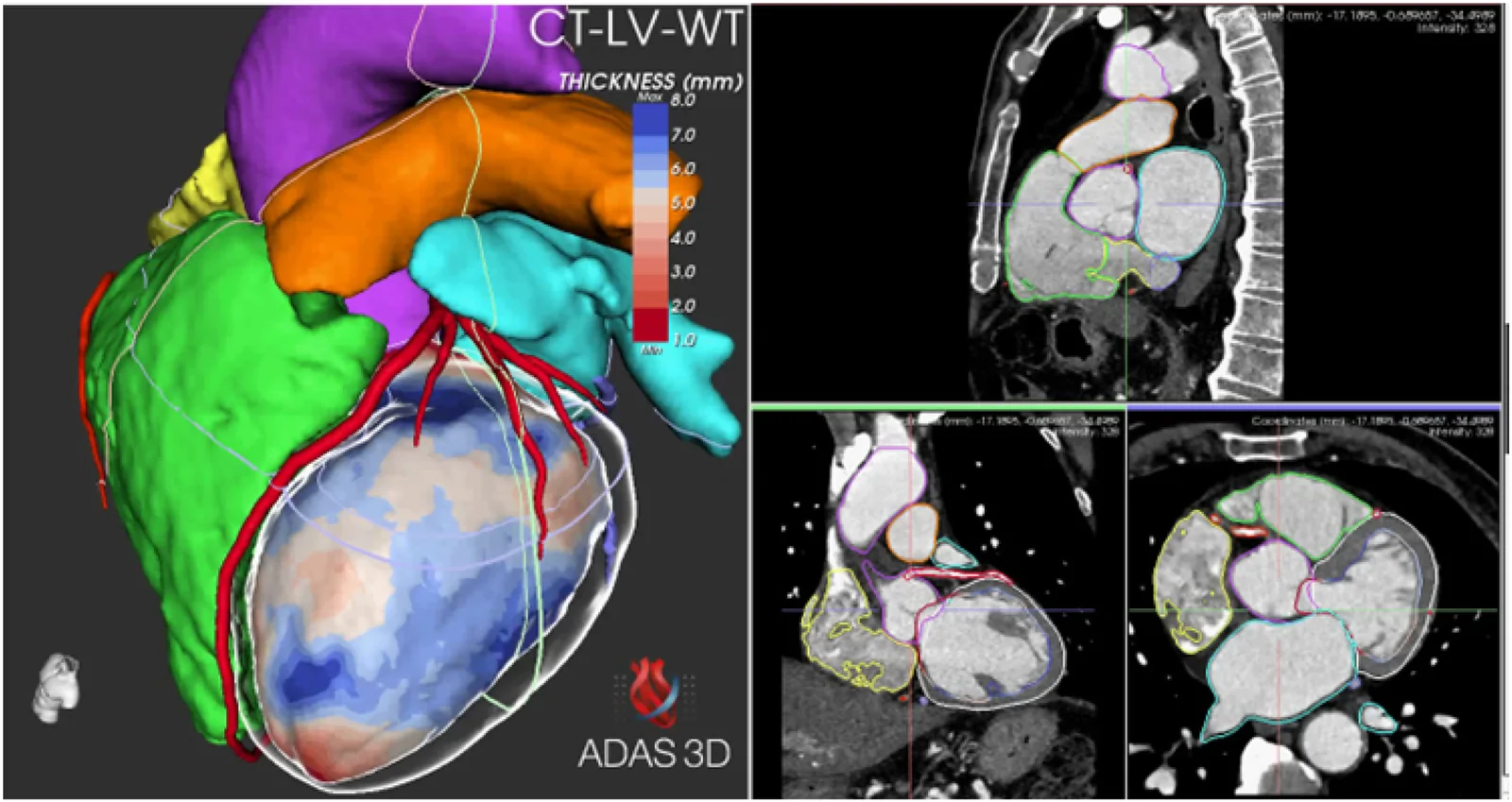
LV wall thickness derived from CT (Image courtesy of ADAS 3D Medical SL, Barcelona, Spain.).

The basis for use of CT is based on the anatomical and functional properties of the tissue. In areas of scar, the wavefront during ventricular tachycardia propagates from area of lesser to greater viable myocardium and the impedance mismatch causes areas of slow conduction creating lines of block. Takigawa et. el., defined CT channels as areas of abnormal thickness (<5 mm), edges thinner at the centre and length greater than width. In a small study of 9 patients VT isthmuses were always found in the CT channels (sensitivity-100 %) with a positive predictive value of 51 percent, particularly those that are longer thinner and exceed 1 mm in depth [32]. (Fig. 3).

The regions of boundaries of the channels are not just anatomical; they are also functional. In a study by Ciaccio et al., high wavefront curvature partially coincided with the preprocedural CT scans and these may be useful in locating the VT isthmus at the infarct border zones [33].

Similar to digital twin for atrial fibrillation, a virtual heart arrhythmia ablation targeting (VAAT) is subject to multisite electrical stimulation protocol to determine the minimum cut required to render the virtual heart non-inducible. Use of virtual heart arrhythmia ablation targeting is another non-invasive approach to determine optimal targets for infarct related ablation. This involves constructing 3D models of the patient's heart from CMR data including both the scar and the border zone and determining optimal ablation sites, using computational modelling, that render the substrate non inducible. This provides with optimal targets on a personalized virtual heart model before the procedure. Using an algorithm and the heart model as a flow network, the smallest amount of tissue which when eliminated, destroys the VT and renders the model non inducible, is calculated. This is then incorporated back in the clinical electroanatomic navigation system. Since this determines the target non-invasively, this reduces the risk of hemodynamic decompensation due to induction of VT during the procedure. It also helps predict the approach to the procedure (endo vs epicardial) which require different anticoagulation strategies and set up. Ablation of VAAT targets resulted in VT non-inducibility and has been described in animal, retrospective and proof of concept prospective patient study, with a small number of patients (n = 5) [34].

Patient undergoing ventricular tachycardia ablation are at high risk for periprocedural hemodynamic decompensation. Traditionally this is predicted by PAINESD score (pulmonary disease, age, ischemic cardiomyopathy, NYHA functional class, ejection fraction, storm, diabetes mellitus), with a score of ≤8 considered low risk, and a score of ≥15 considered as high risk [35]. John et al. proposed addition of scar volume based on the preprocedural CT. CT scar was quantified using predefined attenuation cutoffs: 110-130 HU to represent grey zone tissue and >130 HU to represent dense scar. The highest tertile of scar volume had significantly more unstable complications. When compared to the PAINESD score, a modified PAINES2D classified an additional 2 patients at a higher risk for decompensation [36].

### 4.2 AI-robotics overlap in ablation navigation

Although robotic magnetic navigation has been in use for many years, recently robotic ablation has moved from simple remote control to autonomous navigation. The advantage of magnetic guidance system is that the catheter is gently pulled towards the heart tissue by magnetic force rather than being pushed against it. It allows movement precision which is reproducible, and coupled with a flexible shaft accommodates twists and turns without compromising stability. Robotic magnetic navigation has been combined with VIVO (an AI based non-invasive ECG imaging system) to predict the exact origin of arrhythmia. In a case that had failed with conventional ablation technique, the robotic system used ECGi activation map to determine the ablation target. The case highlights the use of a combination of multiple techniques, AI and robotics to ablate an anterior papillary muscle PVC [37].

#### 4.2.1 Clinical cases

##### 4.2.1.1 Case Vignettes: AI identifies hidden isthmus missed on conventional mapping

In a report by Hojo et el., LUMIPOINT (Boston Scientific) was used to reanalyse a previously reported case where the circuit was presumed to be epicardial. The Rhythmia mapping system includes a confidence mask to grey out areas in which system has a low confidence due to extremely low voltage as in previously ablated areas/diseased tissue and/or due to marked variation in local annotation times. This is normally set to a high value for consistency from the surrounding points. Since the epicardial MB potential is not consistent with the timings to surrounding deflections, this leads to greyed out areas on the mapping system. The focal AT from Marshall bundle (MB) was diagnosed due to the focal centrifugal pattern at the presumed position of MB and from the entrainment data.

This case was reanalysed using LUMIPOINT. By lowering the confidence slider to 20 percent, and the confidence mask level to <0.01 mV (to visualize areas below 0.01 mV) as a scar area, the activation of MB on the epicardium near the termination site could be visualized [38,39].

Similarly, Ho et al., described a case with ventricular tachycardia and recurrent ICD shocks AI-based ECG and CT algorithm was used to localize VT isthmus around left ventricular assist device (LVAD) inflow cannula which is difficult to diagnose due to electromagnetic interference from the LVAD. This was confirmed during activation/voltage and ILAM mapping and ablated [40].

## 5 Limitations

As digital twin and image-derived targeting approaches mature, several guardrails should be recognized to avoid over-interpreting outputs. First, imaging-derived substrate is an imperfect proxy for the true electrical substrate and spatial resolution, partial volume effects, and variability in segmentation thresholds can over- or under-estimate scar and border zones, and device artefact can further degrade reliability. Second, many electrical properties that determine conduction and re-entry are not directly measured noninvasively, so models inevitably rely on assumptions and parameter choices that may not fully capture patient-specific heterogeneity. Third, model outputs can appear precise while uncertainty is substantial. Therefore, simulation-suggested targets should be treated as hypothesis-generating, to be confirmed by mapping and clinical endpoints rather than followed blindly. Fourth, generalizability and reproducibility require standardization and independent validation across centres. Finally, clinical value must be demonstrated beyond technical performance, through workflow feasibility, measurable reductions in procedure burden or risk, and outcomes improvement in adequately powered prospective studies. Recognizing these constraints helps position digital twins as decision support that complements, rather than replaces, operator judgement and invasive confirmation in the EP lab [41].

## 6 Summary

Artificial intelligence has transitioned from a concept to a clinically relevant tool in the EP lab. Automated signal processing addresses the limitations of traditional bipolar mapping while AI assisted CT and MRI processing enables visualization of wall thickness, scar and conducting channels allowing integrating of multimodality imaging to electrophysiological mapping. Emerging digital twin framework extends these capabilities by individualizing patient specific information and virtual testing of ablation strategies.

## 7 Conclusion

Artificial intelligence is slowly emerging as a valuable companion to the clinical care pathways. Availability of large datasets and integration of structured and semi structured data, now, has clinical trial evidence for its use in planning and during ablation of the patient. These are promising especially in patients with complex substrate and promises to bring out newer approaches for these patients. This has also led to emergence of new classification systems based on imaging, use of AI in signal processing and computational modelling [42]. Current AI studies suggest the future EP workflow to evolve to a more data driven and personalized model. This will include preprocedural automated analysis and integration of ECG, CT, MRI and prior electroanatomic maps. This allows the operator to estimate hemodynamic risk, procedural complexity and to plan an individualized ablation strategy. During the procedure, AI assisted signal annotation and automated detection of critical arrhythmogenic substrate such as slow conduction areas or scar will result in improvement of the target selection and shorten mapping time with reduction in inter-operator variability [[43], [44], [45]]. Although there has been an improvement in treatment strategies, significant hurdles remain and these require rigorous validation and development of workflows that allow the various technologies to be integrated efficiently into clinical practice. Continued validation, standardization and careful clinical implementation will be essential to realize the full potential of AI in improving outcomes in electrophysiology lab.

**Table:** **Abbreviation**s

Abbreviation	Expansion
AF	Atrial fibrillation
AI	Artificial intelligence
AT	Atrial tachycardia
CARTOSEG	CARTO Segmentation module
CNN	Convolutional neural network
CT	Computed tomography
DL	Deep learning
ECG	Electrocardiogram
ECGi	Electrocardiographic imaging
EAM	Electroanatomic mapping
EGM	Electrogram
EP	Electrophysiology
FaST	Focal Source and Trigger
FAM	Fast Anatomical Mapping
HU	Hounsfield unit
iAT	iatrogenic atrial tachycardia
ICE	Intracardiac echocardiography
ICD	Implantable cardioverter defibrillator
ILAM	Isochronal Late Activation Mapping
LA	Left atrium
LAWT	Left atrial wall thickness
LGE	Late gadolinium enhancement
LGE-CMR	Late gadolinium enhancement cardiac magnetic resonance
LV	Left ventricle/left ventricular
LVAD	Left ventricular assist device
MDCT	Multidetector computed tomography
ML	Machine learning
MRI	Magnetic resonance imaging
OT	Omnipolar technology
PAINESD	Pulmonary disease, Age, Ischemic cardiomyopathy, NYHA class, Ejection fraction, Storm, Diabetes
PVI	Pulmonary vein isolation
PsAF	Persistent atrial fibrillation
PSI	Pixel signal intensity
PV	Pulmonary vein
RF	Radiofrequency
STAR	Stochastic Trajectory Analysis of Ranked signals
VAAT	Virtual heart arrhythmia ablation targeting
VT	Ventricular tachycardia
VX1	Volta Medical dispersion-mapping software

## Declaration of competing interest

The authors declare that they have no known competing financial interests or personal relationships that could have appeared to influence the work reported in this paper.
